# Six Lectures upon School Hygiene, Delivered under the Auspices of the Massachusetts Emergency and Hygienic Association

**Published:** 1885-10

**Authors:** 


**Six Lectures upon School Hygiene.**—Delivered under the auspices of the Massachusetts Emergency and Hygienic Association to teachers in the public schools. 196 pp., cloth. Price, by mail, 88c. Ginn & Co., Boston.

This little work contains in a nutshell a discussion of all the most important elements of hygiene which have reference to the construction of school buildings and the management of the occupants. The lectures are of a popular character, but the physician cannot fail to be interested in the subjects discussed. The lecture of Dr. F. Folsom upon the relation of our public schools to disorders of the nervous system, is of special interest. This book should be read by all those who are in any way interested in the management of our public schools.

